# Parental Characteristics Associated With Children Born With Neural Tube Defects in Puebla, Mexico

**DOI:** 10.7759/cureus.97046

**Published:** 2025-11-17

**Authors:** Israel Enrique Crisanto-López, Daniela Juárez-Melchor, Aurea Vera-Loaiza, Victoria Sánchez-Muñoz, Damara Guieshuba Vergara-Matus, Mariana Ruiz-Calpe, Alan Alberto Pérez-Arzola, Yazmin Hernández-Castañeda, Oscar Olivares-Huerta, Jonathan Cervantes-Larios, Juan Carlos Flores-Alonso

**Affiliations:** 1 Department of Medical Genetics, General Hospital Zone No. 20, Mexican Social Security Institute, Puebla, MEX; 2 Reproductive Biology Laboratory, Biomedical Research Center of the East, Mexican Social Security Institute, Puebla, MEX; 3 University Center for Health Sciences, University of Guadalajara, Guadalajara, MEX; 4 Department of Medicine, Meritorious Autonomous University of Puebla, Puebla, MEX

**Keywords:** congenital abnormalities, dna fragmentation, neural tube defects, reproductive health, spermatozoa

## Abstract

Background: Neural tube defects (NTDs) are one of the most common congenital anomalies. NTDs are caused by multiple factors that develop in the intrauterine stage and represent a critical birth defect morbidity and mortality. NTDs may depend on a risk factor modifiable by parents; hence, paternal health is important for transgenerational health, as it contributes half of the genetic material for the development of a new individual.

Objective: This study aims to describe demographic, biochemical parameters, and seminal characteristics of parents of offspring with NTDs.

Material and methods: At the General Hospital Zone No. 20 in Puebla, Mexico, parents of newborns or children with NTDs were identified. Clinical, toxicological, and environmental histories were obtained to identify risk factors for NTDs. Additionally, they were requested to provide a semen sample for spermiogram and sperm DNA fragmentation analyses.

Results: Six fathers of children with NTDs were evaluated. Half of the offspring presented with meningocele and half with myelomeningocele. The mean paternal age at conception was 29±5.7 years. None reported folic acid supplementation; 83.3% consumed alcohol, 50% used tobacco, and 66.7% were overweight. Semen analysis showed abnormalities in 50% of cases, and all participants exhibited sperm DNA fragmentation.

Discussion: Paternal preconception health is critically important for offspring development. Exposure to epigenetic factors such as environmental pollution, chemical endocrine disruptors, poor diet quality, lack of physical activity, alcohol and tobacco use, obesity, and conditions that induce oxidative stress can all compromise sperm quality and the health of progeny.

Conclusion: Based on our findings, lack of folic acid supplementation, obesity, alcohol consumption, and sperm DNA fragmentation emerged as significant risk factors for the occurrence of NTDs.

## Introduction

Congenital anomalies are structural or functional alterations that occur in neonates during intrauterine life and can be detected in the prenatal, birth, or infancy period [1]. The causes are multifactorial and may develop due to socioeconomic, demographic, genetic, and environmental factors, as well as a complex interaction among them [2]. Annually, congenital anomalies affect around eight million newborns worldwide and are the leading cause of morbidity and mortality [3].

In fact, congenital anomalies represent a global health problem that particularly affects low- and middle-income countries, which account for 94% of cases, with neural tube defects (NTDs) being one of the most common [4]. In Mexico, the Birth Defects Epidemiological Surveillance System (SVEDAN) reported that during the period 2000-2020, there were 192,273 deaths due to some congenital defect [5]. Additionally, the second quarter of 2025 reported 1,528 birth defects with an incidence of 145.6 cases per 100,000 live births. Among these, the incidence of NTDs was 30.4 cases per 100,000 live births. The main NTDs reported were myelomeningocele, anencephaly, encephalocele, and meningocele [6].

The etiology of NTDs is multifactorial and is mainly associated with genetic, epigenetic, environmental, and nutritional factors, as well as their interactions [7]. Socioeconomic factors predispose individuals to inadequate nutritional states, especially deficiencies of folic acid agonists or inhibitors of dihydrofolate reductase; temporal, regional, or ethnic variations; exposure to teratogens mainly through occupational hazards, substance abuse such as alcoholism and smoking, or medications (primarily antiepileptics, corticosteroids, nonsteroidal anti-inflammatory drugs, acetaminophen, and opioids); as well as comorbidities like obesity, diabetes or immune dysregulation [8-10].

Currently, there are few studies investigating paternal factors associated with the development of NTDs, representing an area of opportunity. Findings in this field will be important for developing more and better preventive strategies that include paternal interventions aimed at family planning through timely genetic counseling for parents, providing care during the periconceptional period, determining possible causes, and performing early and preventive diagnosis that can modify the natural history of NTDs [11].

The present study aimed to describe the age, sociodemographic characteristics, environmental characteristics, folic acid intake, comorbidities and preexisting conditions, anthropometric measurements, biochemical characteristics, and seminal characteristics of parents of offspring with NTDs.

## Materials and methods

Study design and setting

This study was reviewed and approved by the Local Research and Ethics Committee in Health of the General Hospital Zone No. 20 of the Mexican Social Security Institute (IMSS) (registration number R-2023-2108-112). A targeted search was conducted in the consultation records of the Medical Genetics service, and pediatric patients diagnosed with NTDs were selected. Subsequently, the families of these patients were contacted and invited to participate in the study, and a medical consultation was scheduled for those who agreed to take part, where the aim of this study was to collect data and samples. Also, the requirements were eight hours fast for the biochemical panel and sexual abstinence for semen quality analysis.

**Figure 1:**
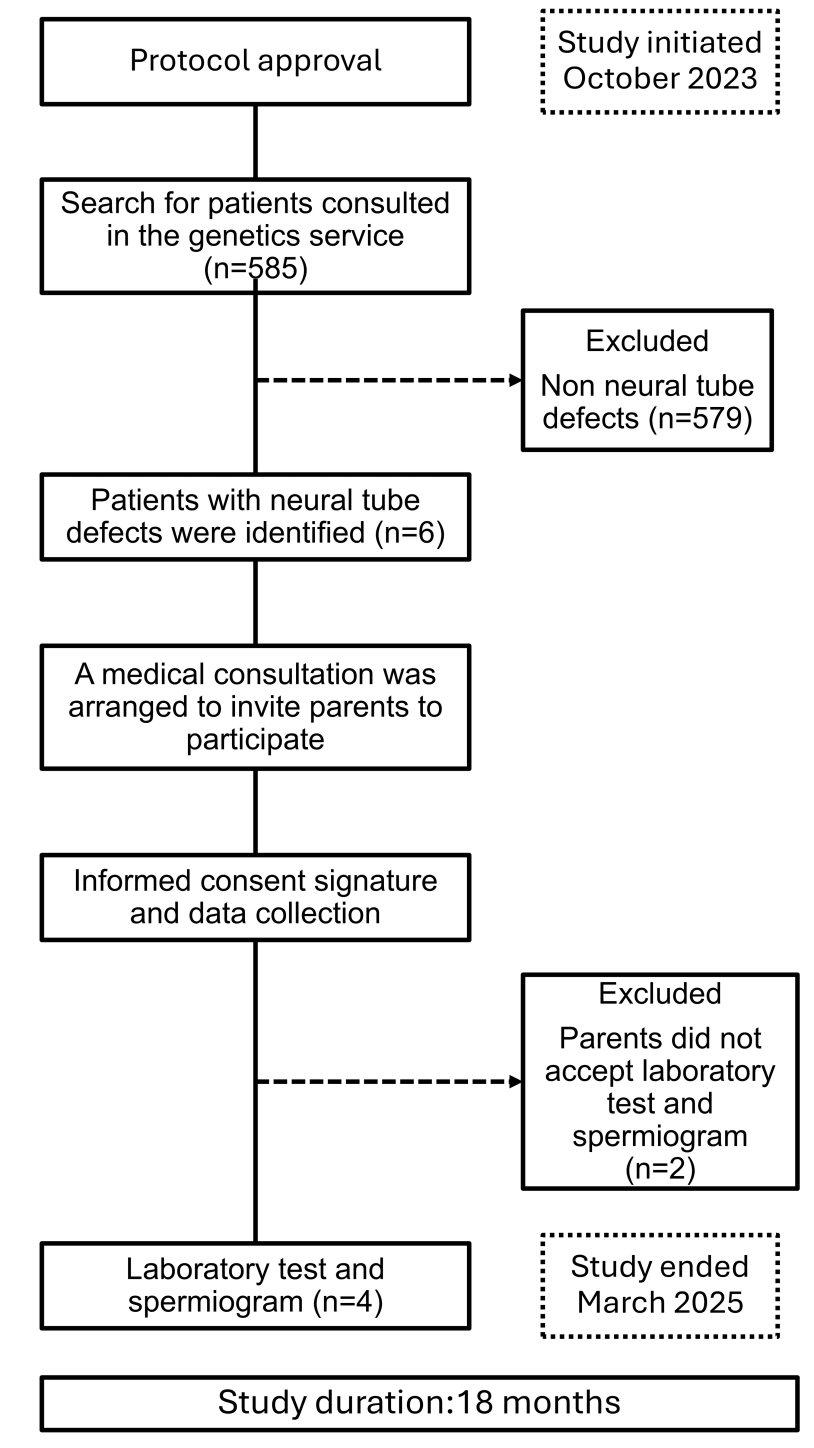
Participant flowchart

The study population consisted of beneficiaries of the IMSS, biological parents of patients who presented NTDs at the General Hospital of Zone No. 20, and who accepted and signed informed consent to participate in the protocol. The sample type was consecutive non-probabilistic, and the study duration was 18 months (Figure 1).

Medical history

The data collected included age, sociodemographic characteristics (place of origin, place of residence, and occupation), environmental characteristics (exposure to medications, alcohol consumption, tobacco use, and comorbidities), folic acid intake, anthropometric measurements, and presence of periconceptional diseases.

Biochemical parameters

Participants were asked to provide a peripheral blood sample for biochemical parameters that included complete blood count and comprehensive metabolic panels. A blood sample was obtained via venipuncture using standard phlebotomy techniques. Samples were collected into appropriate collection tubes and processed according to the hospital’s standard laboratory protocols, and all biochemical analyses were performed using validated methods in accordance with the quality control standards of the clinical laboratory.

Semen quality analysis

Participants were asked to provide a semen sample for sperm analysis (spermiogram) and DNA fragmentation assessment. Patients were instructed to maintain three to seven days of sexual abstinence. Briefly, semen samples were collected in sterile containers and allowed to liquefy at room temperature for 30 minutes. Subsequently, sperm concentration, viability, motility, and morphology were evaluated according to the parameters established in the WHO Laboratory Manual for the Examination and Processing of Human Semen, sixth edition [12]. After initial evaluation, individual samples were washed twice by centrifugation and resuspension at 2,500 rpm for five minutes at room temperature using Tyrode’s albumin lactate pyruvate (TALP) solution (305 mOsm), which contains 114 mM NaCl, 3.2 mM KCl, 25 mM NaHCO_3_, 0.4 mM NaH_2_PO_4_, 10.0 mM sodium lactate, 0.5 mM sodium pyruvate, 2.0 mM CaCl_2_, and 0.5 mM MgCl_2_, pH 7.4, supplemented with 100 mg/L penicillin and 100 mg/L streptomycin. The cells were then resuspended in TALP, counted, and finally adjusted to a concentration of 200,000 spermatozoa/mL.

Assessment of sperm DNA fragmentation by the Comet assay

The Comet assay is a highly sensitive technique that allows quantification of the number of cells with fragmented DNA. The assay is based on the principle that small DNA fragments migrate longer distances toward the anode under an electrophoretic field, forming a characteristic “comet” appearance, whereas larger DNA fragments, such as intact nuclei, remain compact without DNA displacement. Briefly, a base layer of 4% agarose solution was placed on a microscope slide. Then, a second layer composed of a mixture of sperm cells and 3% low-melting-point agarose was added, followed by a third layer of 4% agarose. The preparations were then incubated in an alkaline lysis buffer (2.5 M NaCl, 0.1 M ethylenediaminetetraacetic acid (EDTA), 10 mM Tris(hydroxymethyl)aminomethane (Tris), 1% Triton X-100, 1% dimethyl sulfoxide (DMSO), and 10 mM dithiothreitol, pH 10) for 45 minutes. After lysis, the slides were neutralized with Tris buffer (0.4 M, pH 7.5) and placed in a horizontal electrophoresis chamber where they were subjected to electrophoresis at 300 milliamperes for 15 minutes. Immediately afterward, the slides were washed with Tris buffer (0.4 M, pH 7.5) for neutralization. Next, each slide was stained with Hoechst 33342 solution (0.01 mg/mL) and rinsed with distilled water. Finally, the slides were analyzed using an epifluorescence microscope to determine the number of cells exhibiting nuclear DNA fragmentation, as previously described.

Statistical analysis

All obtained data were analyzed using descriptive statistics according to the nature of each variable. For qualitative variables, frequency and percentage were calculated, while for quantitative variables, measures of central tendency and dispersion were obtained based on the variable distribution after applying the Shapiro-Wilk normality test. Mean and standard deviation were used for variables with a normal distribution; median and interquartile range were used for variables with a non-normal distribution. The analyses were performed using the IBM SPSS Statistics for Windows, Version 25 (Released 2017; IBM Corp., Armonk, New York, United States).

## Results

Parental characteristics of children with NTDs

Data were collected from patients with birth defects who attended the medical genetics service. A total of 585 patients were identified, of whom six had NTDs, representing 1.02% of the population. Subsequently, an appointment was scheduled with each of the six fathers of patients with NTDs. Among these, three cases (50%) involved meningocele and three cases (50%) involved myelomeningocele. The clinical and sociodemographic characteristics of parents are shown in Table 1.

**Table 1:** Parental characteristics of fathers and mothers of children with neural tube defects

Parental Characteristics	Paternal Results	Maternal Results
Current age (average±SD)	31.17±5.30	31.83±6.24
Age at pregnancy (average±SD)	29±5.72	28.5±7.00
Place of origin	4 (66.7%) Puebla, Puebla	4 (66.7%) Puebla, Puebla
	1 (16.7%) Córdova, Veracruz	1 (16.7%) Tehuacán, Puebla
	1 (16.7%) San José Tenango, Oaxaca	1 (16.7%) San José Tenango, Oaxaca
Place of residence	4 (66.7%) Puebla, Puebla	4 (66.7%) Puebla, Puebla
	2 (33.3%) Tehuacán, Puebla	2 (33.3%) Tehuacán, Puebla
Occupation	2 (33.3%) Commerce	5 (83.3%) Housewife
	2 (33.3%) Administrative area	
	1 (16.7%) Textile industry	1 (16.6%) Administrative area
	1 (16.7%) Student	
Drug exposure	1 (16.7%) Bezafibrate use for at least five years prior to conception	No exposure
Folic acid intake	No consumption	Yes, 6 (100%) during the first trimester
Specify folic acid intake	-	5 (83.7%) at 8 weeks of gestation
		1 (16.3%) at 10 weeks of gestation
Alcohol consumption	5 (83.3%)	6 (100%) social use, not during pregnancy
Frequency of alcohol consumption	1 (16.7%) 4 times per month	6 (100%) 5-6 times per year
	1 (16.7%) 3 times per month	
	2 (33.3%) 1-2 times per month	
	1 (16.7%) 5-6 times per year	
Tobacco use	3 (50%)	No consumption
Frequency of tobacco use	1 (16.7%) 3-4 cigarettes per day	-
	1 (16.7%) 1 cigarette every 6 months	
	1 (16.7%) 1 cigarette every year	
Other substance use	No consumption	No consumption
Comorbidities	1 (16.7%) Systemic arterial hypertension and hypertriglyceridemia (5 years of evolution, poorly controlled)	2 (33.3%) Obesity
SARS-COV-2 infection	2 (33.3%)	No infection
Confirmed COVID-19	No confirmed	No confirmed
Height (m) (media±SD)	1.70±0.09	1.56±0.03
Weight (kg) (media±SD)	76.33±10.17	63.90±19.81
BMI (media±SD)	26.09±2.50	26.21±7.90
BMI classification	4 (66.7%) Overweight	4 (66.7%) Healthy weight
	2 (33.3%) Healthy weight	1 (16.6%) Class 1 obesity
		1 (16.6%) Class 2 obesity

Biochemical parameters of patients

The biochemical parameters of patients are presented in Table 2. Alterations were observed in glucose levels (119.32 ± 45.88 mg/dL), total cholesterol (181.3 ± 46.48 mg/dL), triglycerides (239.45 ± 113.05 mg/dL), and very-low-density lipoproteins (VLDLs) (50.05 ± 22.42 mg/dL). In the urinalysis, only one participant showed abnormal findings, including the presence of proteins, glucose (300 mg/dL), blood (50 erythrocytes/µL), leukocytes, bacteria, mucin threads, calcium oxalate, and amorphous urate crystals. In the complete blood count, no abnormalities were observed in the reported parameters.

**Table 2:** Biochemical parameters of fathers with children NTD diagnosed ^†^ Values exceeding the reference range. HDL: high-density lipoproteins; LDL: low-density lipoproteins; VLDL: very-low-density lipoproteins; NTD: neural tube defect

Complete Blood Count			
Parameters	Results (average±SD)	Units	Reference values
Red blood cells	5.14±0.21	x10^6^/µL	4.59-6.50
Hemoglobin	16.1±0.85	g/dL	13.8-16.7
Hematocrit	46.2±3.47	%	40.5-52.0
Platelets	183±54	x10^3^/µL	150-450
White blood cells	4.81±0.65	x10^3^/µL	4.8-10.0
Metabolic Panel			
Parameters	Results (average±SD)	Units	Reference value
Glucose	119.32±45.88^†^	mg/dL	85-100
Urea	29.49±1.92	mg/dL	16.60-49.50
Uremic nitrogen	13.75±0.95	mg/dL	8.00-23.00
Creatinine	1.04±0.12	mg/dL	0.50-1.20
Total cholesterol	181.3±46.48^†^	mg/dL	140-220
Triglycerides	239.45±113.05^†^	mg/dL	35-150
HDL	50±8.08	mg/dL	45.00-60.00
LDL	89.04±28.8	mg/dL	<100
VLDL	50.05±22.42^†^	mg/dL	2-30

Sperm analysis

**Figure 2:**
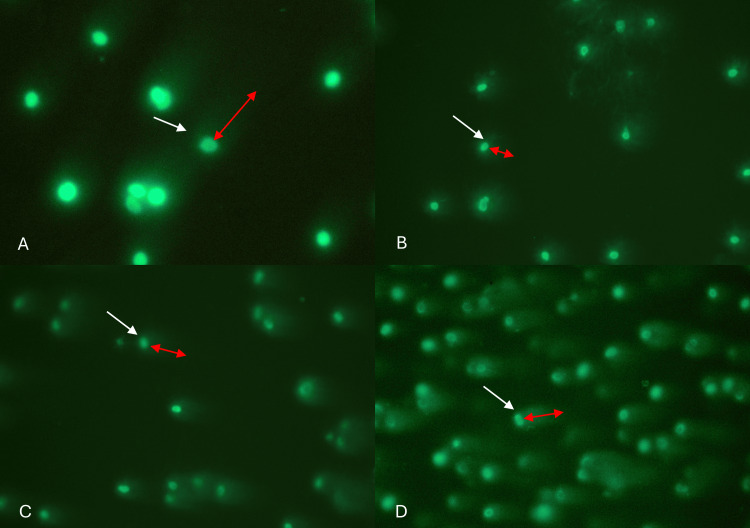
Comet assay is a technique based on single-cell electrophoresis used to detect DNA fragmentation and its extent. Cells without DNA fragmentation appear as intact nuclei and the cells with DNA fragmentation show nuclei surrounded by halos and a tail-like streak resembling a comet (white arrow); the degree of fragmentation is indicated by the distance the DNA migrates from the nucleus (red arrow). (A) Sperm cells with DNA fragmentation from patient 1, (B) patient 2, (C) patient 3, and (D) patient 4.

Sperm analysis (spermiogram) and sperm DNA fragmentation testing were performed on only four fathers whose children were diagnosed with meningocele (n=1) and myelomeningocele (n=3); two individuals declined to provide a semen sample. Among the analyzed samples, three exhibited normozoospermia, while two samples (50%) showed oligonecroteratozoospermia and necrozoospermia (Table 3). Furthermore, all samples demonstrated sperm DNA fragmentation in more than 30% of sperm cells from the fathers of patients with NTDs (Figure 2).

**Table 3:** Spermiogram analysis results of fathers with children NTDs diagnosed ^†^ Values exceeding the reference range. Reference values according to the WHO Laboratory Manual for the Examination and Processing of Human Semen, 6th Edition, 2021. NTD: neural tube defect

Parameter	P1 (40 Years Old)	P2 (26 Years Old)	P3 (31 Years Old)	P4 (26 Years Old)	Reference Value
Volume	3.5	3.8	1.5	2	1.4 (1.3-1.5) mL
Color	Yellow	Yellow	Opalescent gray	Opalescent gray	White-grayish
Coagulum	Absent	Present	Present	Present	Present
Liquefaction	60	60	60	60	5-60 minutos
Viscosity	Increased	Increased	Increased	Increased	Normal
Appearance	Opalescent	Opalescent	Opalescent	Opalescent	Translucent-opalescent
pH	8	8	8	8	7.2-8.0
Sperm concentration	0.208^†^	213.8	313	90.5	16.0 (15-18) mill/mL
Total sperm count	0.728^†^	812.44	469.5	181	39 (35-40) millons
Sperm motility	36.19	92.71	94.9	60.48	30 (29-31)%
Sperm viability	11.36^†^	70.17	74	43.55^†^	54 (50-56)%
Sperm morphology	0.99^†^	50.32	43.6	30.64	4 (3.9-4.0)%
Sperm DNA fragmentation	97.87^†^	69.68^†^	94.94^†^	100^†^	<30%
Finding	Oligonecroteratozoospermia	No abnormalities detected	No abnormalities detected	Necrozoospermia	No abnormalities detected
NTD type	Mielomeningocele	Mielomeningocele	Meningocele	Mielomeningocele	-
Observations	Hypertriglyceridemia >5 years and bezafibrate consumption	Consumed alcohol	Consumed alcohol and smoked tobacco	Consumed alcohol and smoked tobacco	-

## Discussion

Worldwide, NTDs represent a public health problem as they cause morbidity and mortality in newborns. In Mexico, the incidence of NTDs has shown an increasing trend in recent years, with a reported rate of 33.5 per 100,000 live births in 2024. Medical care for these patients is provided at secondary and tertiary levels of care, depending on the type of NTD, age at diagnosis, and complications presented [6,13]. In this study, despite being conducted at a secondary-level hospital, only three patients with meningocele and three patients with myelomeningocele were identified during the study period. These patients were referred to and received care in the Department of Medical Genetics service.

The etiology of NTDs is multifactorial and includes genetic and environmental factors; their pathophysiology is not yet fully understood. Gene-gene, gene-environment, gene-nutrient interactions, and neuroinflammation have been described [7,14]. The genetic mechanisms that regulate neural tube closure are complex and controlled by the interaction of various genes, such as *BMP, Wnt, SHH, FGF, TGF-β*, and environmental factors [15-17]. It has been proposed that the *MTHFR* C677T gene variant, which encodes the thermolabile enzyme methylenetetrahydrofolate reductase, increases the risk of NTDs [18,19]. Studies show that polymorphisms in the paternal *MTHFR* gene are mostly associated with pregnancy loss, fertility problems, and congenital anomalies, and it is even suggested that folic acid and vitamin B12 supplementation be part of preconception planning [20,21]. Additionally, it has been shown that folic acid supplementation has beneficial effects on seminal parameters, sperm DNA fragmentation, and pregnancy development in patients with oligozoospermia and *MTHFR* gene polymorphisms [22].

Mexico has reported cases of anencephaly associated with *MTHFR* gene polymorphisms in women from the states of Mexico, Guerrero, and Puebla [23,24]. Among our study population, we observed that 66.7% of the fathers and mothers were originally from Puebla, suggesting that this demographic factor may confer a genetic predisposition related to *MTHFR* gene variants.

Few studies focus on linking paternal factors as risks for the development of NTDs. Studies show that paternal age [25-27], geographic area, place of residence of the parents [28], environmental conditions (chemical exposure, radiation, and pollution such as pesticides, solvents, and paints), occupation (painters, printing or vehicle operators, workers in plastic production, farmers, food, beverage, wood or textile processing industries, and those engaged in activities with exposure to ionizing radiation) [29-31], folic acid deficiency [7-10] increase the risk of developing an NTD [32-34].

Paternal age beyond the optimal fertility window (20-45 years) has been associated with adverse effects on offspring health [25,35,36]. In our study, the fathers were within the optimal fertility window. Likewise, residing in Puebla could suggest that environmental agents predispose to NTDs, such as contaminants from rivers and lakes, including heavy metals, polycyclic aromatic hydrocarbons, or pesticides [37,38]. However, none of the patients studied reported having lived in areas close to rivers or lakes.

Exposure to teratogens also contributes to the development of NTDs. In our study, 83.3% of fathers reported alcohol consumption, and 50% reported tobacco use. Both habits have been described as compromising the regulatory mechanisms involved in neurulation processes [39,40]. Alcohol interferes with folic acid transport, while tobacco use reduces the expression of NOG, a gene specifically expressed in the caudal region that promotes closure of the posterior neuropore. Additionally, tobacco use negatively affects the secretory function of Sertoli cells, induces oxidative stress in testicular tissue, reduces circulating testosterone and gonadotropins, causes testicular shrinkage, and disrupts spermatogenesis [41]. Moreover, smoking leads to the overproduction of reactive oxygen species and oxidative stress. Cigarette smoke contains cadmium, which can induce DNA strand breaks, and its consumption has been associated with congenital heart defects, limb anomalies, and NTDs in offspring [42,43]. Studies have shown that the use of harmful substances is associated with offspring abnormalities such as birth defects, mental health disorders, hyperactivity, and attention-deficit/hyperactivity disorder [44,45]. It is worth noting that these factors (smoking, alcohol consumption, and exposure to chemical agents or medications) have also been linked to an increased risk of congenital heart defects [46].

The habitual use of medications such as antiepileptics, corticosteroids, non-steroidal anti-inflammatory drugs, paracetamol, or opioids has also been associated with an increased risk of developing NTDs [7,47]. In our study population, only 16.7% of participants reported a history of bezafibrate use for a period of five years, prescribed due to hypertriglyceridemia of five years’ duration without adequate metabolic control. In murine models, bezafibrate may regulate tau protein expression, cerebral glucose metabolism, and neuroinflammation, exerting neuroprotective effects [48]. Additionally, bezafibrate improves spermatogenesis affected by type 2 diabetes, and it has even been proposed as a potential strategy for treating male infertility [49]. Despite these reported benefits, emphasis is placed on the importance of achieving proper lipid control in patients [50]. In this regard, obesity, diabetes, and immune dysregulation have been shown to be associated with the development of NTDs [7,9]. In our study, the fathers' BMI was classified as overweight (26.09 ± 2.50 kg/m^2^), which is a known predisposing factor for obesity and, consequently, for possible fertility disorders and congenital anomalies such as NTDs, as previously suggested.

NTDs can result from modifiable risk factors, such as folic acid deficiency. In Mexico, during 2025, the Epidemiological Surveillance Directorate for Non-communicable Diseases reported that 29% of mothers did not take folic acid or began supplementation after the first trimester of pregnancy. Additionally, 96.2% of fathers did not take folic acid [6]. Moreover, a link has been demonstrated between folate deficiency in sperm and the development of NTDs such as spina bifida [25,51,52]. In our study, none of the fathers reported preconceptional folic acid supplementation. All of them (100%) stated that the reason was a lack of awareness and the absence of a medical recommendation. In contrast, 100% of the mothers who reported taking folic acid did so under medical advice at the time of pregnancy recognition. These findings highlight the need to promote folic acid supplementation among fathers and to strengthen support for male reproductive health [33,53].

On the other hand, the damage caused by the SARS-CoV-2 virus continues to be reported. Studies have documented an association between maternal SARS-CoV-2 infection and the development of meningocele in the sacral region [54,55]. Regarding males, it has been observed that the infection tends to be acute when there is high expression of *ACE2* and *TMPRSS2* in the testes, inducing inflammatory and hypoxic processes that interfere with testosterone production and alter the permeability of the blood-testis barrier [56]. In our study, 33.3% (n=2) of the fathers reported SARS-CoV-2 infection during the periconceptional period, although none developed COVID-19 disease. It has been described that SARS-CoV-2 may cause testicular damage by entering testicular cells through binding of the viral spike glycoprotein (S) to ACE2, while the TMPRSS2 facilitates membrane fusion between the virus and host cells. This mechanism may result in impaired male fertility, decreased sperm quality, and even sperm DNA fragmentation, thereby increasing the risk of congenital anomalies such as NTDs [57-59].

Male fertilizing capacity is evaluated based on sperm function and quality, typically assessed through spermiogram analysis and sperm DNA fragmentation tests. Of the six fathers of patients with NTDs included in the study, only four agreed to proceed with semen analysis. Among this subgroup, 50% were diagnosed with oligonecroteratozoospermia and necrozoospermia. Additionally, 100% of the samples analyzed showed a high percentage of sperm with DNA fragmentation, with individual values of 97.87%, 69.68%, 94.94%, and 100%. Previous studies have shown that paternal age, testicular function, and reproductive hormones can affect sperm parameters and DNA integrity, as well as telomere length, de novo mutation rates, chromosomal structure, and epigenetic factors [35].

Paternal preconception health is critically important for offspring development. Exposure to epigenetic factors such as environmental pollution, chemical endocrine disruptors, medications, as well as poor diet quality, lack of physical activity, alcohol and tobacco use, obesity, trauma, and conditions that induce oxidative stress, can all compromise sperm quality and the health of the progeny [60].

Epigenetic mechanisms have been proposed to influence sperm quality through modifications of sncRNA [61-63]. Studies suggest that paternal folate deficiency can alter the sperm epigenome via DNA methylation [64,65]. Additionally, sperm quality can be impaired even in normozoospermic individuals due to damage to sperm DNA, which may be affected by apoptosis, DNA strand breaks, alterations in reactive oxygen species production, increased activity of endogenous caspases and endonucleases, exposure to radiotherapy and chemotherapy, as well as environmental toxins [66]. Sperm quality is assessed through sperm DNA fragmentation, which refers to breaks or damage in the DNA strands within the sperm nucleus, and is reported as the percentage of cells exhibiting such damage [67].

Alterations in paternal metabolism are associated with variations in sperm quality. In our study, we observed elevated levels of glucose, cholesterol, triglycerides, and VLDL (Table 2). This may be detrimental, as glycemic susceptibility has been linked to erectile dysfunction, male infertility, and abnormal sperm production [68,69]. Furthermore, in male mice, a high-cholesterol diet has been shown to significantly increase atherosclerosis only in female offspring and to alter gene expression related to non-coding RNA biogenesis in the epididymis - but not in the testes - which may impair sperm motility [70].

Triglycerides, as the main source for steroidogenesis, are critically important for spermatogenesis through the lipid peroxidation reaction, which produces free radicals and reactive oxygen species that damage polyunsaturated fatty acids in the cell membrane. Hypertriglyceridemic states harm the plasma membrane and impair sperm function by reducing motility and hindering sperm-oocyte fusion. Additionally, lipid peroxidation products can react with DNA, leading to genotoxicity [71]. Likewise, VLDL influences serum lipid concentrations and has been associated with a higher number of sperm cells showing DNA fragmentation [72,73]. Moreover, paternal metabolic syndrome during the periconceptional period has been associated with an increased risk of congenital anomalies in offspring [74]. In this study, we found that even when spermiogram results fall within normal reference parameters, this does not rule out the possibility of compromised sperm DNA integrity. Therefore, it is crucial to recognize and prioritize male reproductive health and to strengthen periconceptional care and family planning strategies in the general population by promoting healthy lifestyles and preventive measures.

In this regard, one of the main strengths of this research lies in addressing male reproductive health as a complementary and important factor in the development of NTDs, a topic that remains underexplored in the current scientific literature. This represents a unique opportunity to contribute new knowledge to an emerging field of study. Its relevance is heightened by the growing need to develop preventive strategies for public health issues such as NTDs and congenital anomalies.

At the same time, we acknowledge that one of the limitations of this study is the small number of patients included, which we attribute to multiple factors. These include the lack of proper follow-up by the entire healthcare team, particularly the referral of patients with NTDs to the genetics department for further evaluation to rule out syndromic conditions, provide genetic counseling, and assess recurrence risks. Moreover, this raises important questions about real-world access to healthcare systems, especially for patients from remote communities or those who lack adequate prenatal and periconceptional care. Timely reporting to national registration and surveillance systems also remains a challenge, ultimately hindering accurate estimation of the true prevalence of NTDs.

Additionally, raising awareness about the importance of male reproductive health and promoting optimal preconception conditions for fathers through clinical consultation, as well as biochemical parameters, semen analysis, and sperm DNA fragmentation testing, is essential. It is also necessary to overcome barriers such as cultural, social, and personal stigma, taboos surrounding sexuality, and widespread misinformation. Nevertheless, despite these challenges, this study remains relevant as it provides valuable insights for further investigation into the role of paternal factors and male reproductive health - not only in relation to NTDs but also in other congenital disorders. This contribution may help foster greater interest, awareness, and expanded understanding of the topic in the future.

## Conclusions

The absence of folic acid supplementation, obesity, alcohol consumption, and compromised sperm DNA integrity were the paternal factors observed in the fathers of children with NTDs in the patient population of the General Hospital Zone No. 20. Additionally, paternal alcohol and tobacco use, along with comorbidities such as overweight, may induce biochemical alterations, including elevated glucose, total cholesterol, triglycerides, and VLDL levels, which could be associated with the development of NTDs.

Therefore, paternal reproductive health is vital for proper conception, growth, and birth, highlighting a critical period for the future health of the offspring. This research project opens an area of opportunity for the prevention of NTDs, which are multifactorial conditions linked to paternal reproductive health.
